# Electrohysterography for Uterine Contractility Monitoring: Measurement Principles, Clinical Evidence, and Reporting Recommendations

**DOI:** 10.3390/s26154669

**Published:** 2026-07-23

**Authors:** Gulnur Bayramli, Koushita Gouri Reddy Valluru, Ravi Goyal

**Affiliations:** Department of Obstetrics and Gynecology, College of Medicine, University of Arizona, Tucson, AZ 85724, USAkoushitav@arizona.edu (K.G.R.V.)

**Keywords:** electrohysterography, uterine electromyography, cardiotocography, uterine contraction monitoring, preterm birth, wearable sensors, biomedical signal processing, reporting standard

## Abstract

Reliable monitoring of uterine contractility underpins the assessment of labor, the diagnosis of preterm labor, and the timing of obstetric intervention, yet routine methods measure only the mechanical consequences of contraction. External tocodynamometry and cardiotocography (CTG) are operator- and position-dependent and perform poorly with maternal obesity, detecting as few as ~54% of the contractions confirmed by an intrauterine pressure catheter, whereas surface electrohysterography (EHG) detects upward of ~94% by recording the myometrial electrical activity that drives contraction. This review examines EHG and uterine electromyography (EMG) as measurement modalities, covering their physiological origin, acquisition hardware, signal characteristics, feature extraction, and machine-learning analysis, and compares them with CTG against the intrauterine pressure catheter reference standard. Electrical approaches additionally yield predictive parameters, notably spectral peak frequency and propagation velocity, that mechanical methods cannot provide. The principal barrier to translation is methodological heterogeneity rather than physiology: differences in electrodes, filtering, feature definitions, and outcome measures preclude cross-study comparison and meta-analysis. As its central contribution, this review consolidates prior calls for standardization into a minimum reporting set for EHG studies and appraises translational readiness, identifying prospective external validation, shared datasets, explainable models, and outcome-linked trials as priorities.

## 1. Introduction

Monitoring uterine contractility is crucial in obstetrics for assessing labor progression, diagnosing preterm labor risk, and guiding interventions. Traditionally, uterine activity has been monitored indirectly via mechanical methods such as external tocodynamometry or the intrauterine pressure catheter (IUPC). External tocodynamometry senses only the tension the contracting uterus exerts on the abdominal wall, not intrauterine pressure, while the intrauterine pressure catheter measures pressure invasively; neither captures the myometrial electrical activation that drives contraction. Cardiotocography (CTG), the most widely used clinical modality, combines Doppler ultrasound assessment of fetal heart rate with tocodynamometric recording of uterine activity, but it likewise infers contractions from mechanical signals rather than measuring myometrial physiology directly.

In recent decades, bioelectrical approaches have gained attention, particularly the electrohysterogram (EHG), a noninvasive method that records the electromyographic (EMG) activity of uterine muscle from the surface of the abdomen. These methods capture the electrical impulses generated by uterine smooth muscle cells during contractions, offering a more direct look into the physiology of labor.

A dedicated, measurement-focused synthesis of these modalities is both timely and needed. Preterm birth complicates roughly one in ten pregnancies and remains the leading cause of neonatal death and long-term childhood disability, yet the tools used clinically to detect and characterize the uterine contractions that precede it remain strikingly limited. External tocodynamometry and cardiotocography register only the mechanical deformation of the abdominal wall; they are operator- and position-dependent, perform poorly in the rapidly growing population of patients with obesity, and miss roughly half of the contractions confirmed by an intrauterine pressure catheter, while the catheter itself is invasive and confined to active labor [1,2]. EHG and uterine EMG address this gap by measuring the myometrial electrical activity that actually drives contraction, and they additionally yield predictive parameters, such as spectral peak frequency and propagation velocity, that mechanical methods cannot provide [3]. Over the past decade, progress in electrode design, signal processing, and machine learning has been rapid but fragmented across engineering and clinical literatures, and the recent regulatory clearance of wearable EHG monitors together with the expansion of remote prenatal care has moved uterine electrophysiology from the laboratory toward routine use. These developments create an urgent need for a single reference that consolidates the physiological basis, harmonizes terminology, integrates the analytical and clinical evidence, and identifies the standardization and validation gaps that still impede adoption. This review is intended to serve that purpose, and it makes four contributions. First, it provides a measurement-oriented account of EHG and uterine EMG that follows the signal from its physiological origin through acquisition hardware, feature extraction, and machine-learning analysis. Second, it offers a structured, parameter-by-parameter comparison of EHG, invasive or internal uterine EMG, and CTG against the intrauterine pressure catheter reference standard (Table 1). Third, as its central methodological contribution, it proposes a minimum reporting standard for EHG studies (Table 2) that operationalizes the field’s standardization gap into concrete, checkable items spanning acquisition, signal processing, and clinical validation, thereby addressing the heterogeneity that currently impedes reproducibility, regulatory qualification, and meta-analysis. Fourth, it appraises the translational readiness of EHG, distinguishing applications that are clinically supported, promising, and experimental, and sets out a prioritized, trial-oriented research agenda.

Scope and approach: This is a narrative topical review rather than a systematic review or meta-analysis. We identified relevant literature through searches of PubMed, IEEE Xplore, and Scopus from database inception to June 2026, combining the terms “electrohysterography,” “uterine electromyography,” “EHG,” “uterine EMG,” “cardiotocography,” “tocodynamometry,” “preterm labor,” and “uterine contraction monitoring,” and supplementing them with backward citation tracking of key articles and reviews. Priority was given to foundational physiological studies, methodological and signal-processing developments, and clinical validation studies, with emphasis on peer-reviewed primary research and authoritative reviews. Because acquisition protocols, feature definitions, study populations, and outcome measures vary widely across this literature, we did not attempt quantitative pooling; instead, we synthesize the evidence qualitatively and, in the closing sections, propose a reporting framework intended to make future quantitative synthesis feasible. Recent reviews have surveyed advances in EHG signal processing, machine learning, and clinical application [4,5].

This review examines, in turn, the development, physiological basis, acquisition, signal characteristics, analysis, clinical applications, and limitations of EHG and uterine EMG; introduces CTG as the current clinical standard; and presents a structured three-modality comparison. It then surveys emerging wearable technologies, proposes a minimum reporting standard, and sets out a prioritized research agenda.

## 2. Electrohysterography (EHG)

Electrohysterography refers to the recording of uterine electromyographic signals from the body surface. Functionally, it represents a surface EMG of the uterus, obtained noninvasively via electrodes placed on the surface of the abdomen. Throughout this review, EHG denotes this noninvasive, surface form of uterine EMG; “invasive uterine EMG” is reserved for internal (intrauterine or intramyometrial) recordings used only in research, and all clinical and home applications discussed here use surface EHG. Below, we review the development, physiological underpinnings, technical aspects, signal properties, analytical techniques, clinical applications, and challenges of EHG.

### 2.1. Historical Development and Context

The concept of measuring uterine electrical activity from the abdominal surface emerged in mid-twentieth-century obstetric research. Early pioneers such as Steer and Hertsch first recorded electrical signals from the human uterus during labor in 1950, coining the term “electrohysterograph” for this technique [6]. Subsequent studies from the period 1950–1970 confirmed that bursts of bioelectrical activity accompany uterine contractions. For example, Larks and Dasgupta (1958) characterized EHG waveforms during pregnancy and labor [7], and Wolfs and van Leeuwen (1979) performed detailed electromyographic observations on the human uterus throughout labor [8].

By the 1980s, researchers had refined external uterine EMG recording techniques. Planes et al. (1984) demonstrated improved methods to capture the fast electrical activity of the uterus noninvasively during parturition [9]. These foundational works established that uterine contractions are accompanied by reproducible electrical signals detectable on the abdominal surface. In 1993, Devedeux and colleagues published a critical review synthesizing animal and human uterine EMG findings, solidifying electrohysterography as a viable modality for monitoring pregnancy and labor [10]. Since then, numerous studies have advanced EHG technology and analysis, aiming to develop it into a clinical tool for labor monitoring and preterm-birth prediction.

### 2.2. Physiological Basis of the EHG Signal

Uterine contractions originate from the coordinated electrical activation of the myometrium (uterine smooth muscle). Unlike skeletal muscle, which is governed by somatic motor neurons and organized into motor units, uterine smooth muscle is hormonally primed and intrinsically excitable, especially as pregnancy progresses. The physiological basis of the EHG lies in the generation and propagation of uterine smooth muscle action potentials.

Myometrial electrical activity: Uterine myocytes generate spontaneous depolarizations. During pregnancy, rising estrogen and declining progesterone levels near term increase expression of ion channels and gap junctions (connexin-43) in the myometrium, enhancing excitability and electrical coupling between cells [11]. These changes create a syncytium that can support long-range propagation of electrical signals. Action potentials in uterine muscle typically manifest as bursts of rapid spikes (from Ca^2+^-mediated currents), often superimposed on a slow depolarizing plateau. The synchronized bursts across many fibers produce a compound electrical signal that correlates with a contraction.

Pacemakers and propagation: The uterus does not have a single pacemaker region; any sufficiently depolarized area can initiate a contraction. Classic studies have established that effective labor requires a rapid, near-synchronous spread of action potentials throughout the uterus, enabled by intercellular coupling through gap junctions [12]. This near-simultaneous contraction of the upper uterine segments differs from the orderly wave propagation seen in organs such as the heart. Electrical impulses in the uterus propagate via cell-to-cell coupling (gap junctions) at a relatively slow conduction velocity, on the order of a few centimeters per second. During active labor, high connectivity through gap junctions allows the uterus to behave as a coordinated electrical syncytium. Garfield and Hayashi’s seminal work showed that the appearance of abundant gap junctions in human myometrium is strongly correlated with the onset of labor, enabling synchronized electrical activity and effective contractions [12].

In summary, the uterine EHG signal originates from the summated action potentials of thousands of smooth muscle cells firing in concert, with signal propagation and amplitude modulated by the degree of cellular excitability and coupling present.

### 2.3. Signal Acquisition Techniques for EHG

**Figure 1:**
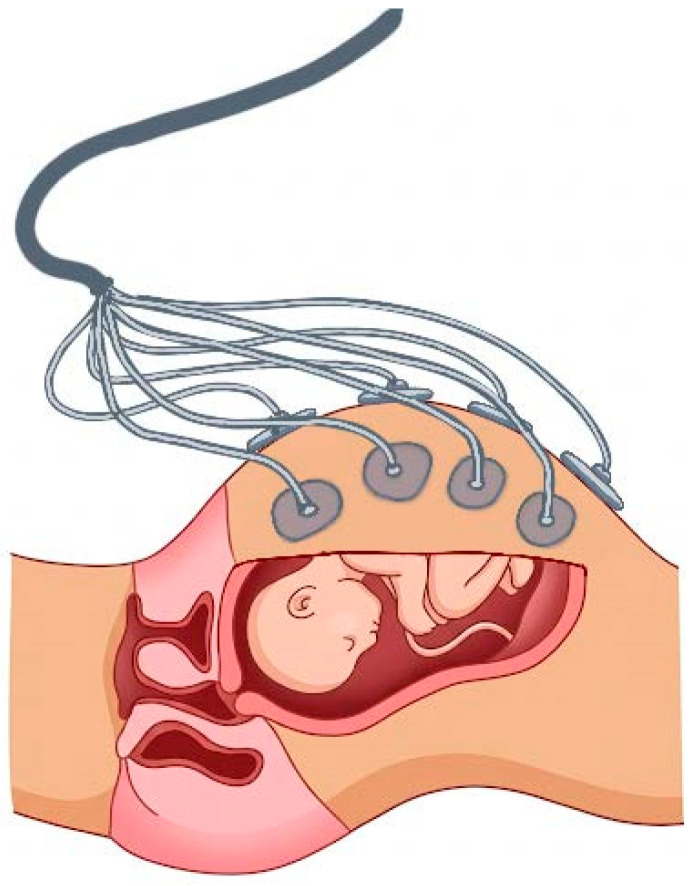
Noninvasive surface electrohysterography. A multi-electrode array of adhesive Ag/AgCl electrodes is placed on the maternal abdomen overlying the uterus, recording the summated myometrial electrical activity transmitted through the abdominal wall.

Electrode types and placement: EHG acquisition is noninvasive and uses surface electrodes, similar to those employed in electrocardiography (ECG), to capture uterine electrical activity. Disposable Ag/AgCl adhesive electrodes are placed on the maternal abdomen overlying the uterus. Various electrode configurations have been used: common setups include a set of bipolar electrodes positioned around the maternal navel or lower abdomen, or multi-electrode arrays (e.g., a grid of electrodes) to capture spatial information. For instance, a standardized 4 × 4 electrode array (16 channels) placed across the abdomen has been employed in research to map uterine electrical activity patterns [13]. Electrode spacing ranges from about 2.5 to 7 cm between bipolar pairs in different studies [10]. A reference electrode (ground) is placed on an electrically neutral point (e.g., the hip) to reduce noise (Figure 1).

Hardware and amplification: The raw uterine EMG signals are low in amplitude and frequency, necessitating specialized biopotential amplifiers. High-input-impedance differential amplifiers with good common-mode rejection are used to amplify the EHG while minimizing interference from external noise and physiological artifacts. Signals are typically amplified on the order of 1000 to 10,000 times. To prevent aliasing, sampling rates between 100 and 500 Hz are generally selected, which adequately cover the uterine EMG spectrum (<5 Hz).

Filtering: Signal filtering is critical for isolating uterine activity. A band-pass filter is commonly applied: the high-pass cutoff is often set around 0.1 Hz to remove baseline drift caused by respiration or electrode motion, while the low-pass cutoff is typically 3 to 5 Hz to capture the dominant “fast wave” activity while suppressing maternal ECG and higher-frequency noise. Some studies extend the low-pass limit up to 100 Hz for detailed EMG analysis, though for routine monitoring, uterine signal energy remains concentrated below 5 Hz. Powerline interference at 50/60 Hz can be further attenuated with notch filters. Practical measures such as skin preparation (light abrasion, conductive gel) and careful electrode fixation also reduce artifacts. Bipolar or figure-of-eight configurations help cancel common-mode noise, improving signal fidelity.

Multichannel and Laplacian techniques: Beyond single-channel recordings, multichannel EHG provides spatial information and supports advanced processing. Surface Laplacian methods can enhance local activity and suppress distant interference, thereby improving the signal-to-noise ratio [13]. Portable systems have further advanced the field; for instance, the Monica AN24 device uses five abdominal electrodes (four active and one ground) to simultaneously record fetal ECG and uterine EMG, transmitting data wirelessly for remote monitoring [14]. Recent innovations in adhesive electrode patches and compact wireless amplifiers have enabled long-duration monitoring in both clinical and home environments.

### 2.4. Signal Characteristics and Typical EHG Profiles

Uterine EMG signals have distinctive characteristics in both the time and frequency domains. Key features include amplitude, frequency content, temporal pattern, and propagation.

Amplitude: The EHG is a low-amplitude signal. During uterine contractions, the peak-to-peak amplitude typically ranges from a few tens of microvolts up to about 1 millivolt on the abdominal surface. Studies report that the higher-frequency “fast” component of EHG usually has amplitude under 1 mV, whereas the low-frequency “slow” baseline shift can reach several millivolts [10]. As labor progresses, the EHG amplitude tends to increase due to stronger and more synchronized contractions. However, absolute amplitude can vary widely between patients and with electrode placement, so amplitude alone is not a specific indicator of labor unless normalized within-subject.

Frequency content: Two primary spectral components have been identified. The slow wave (SW) is a very-low-frequency component (0.01–0.1 Hz) reflecting baseline shifts related to uterine tonus or abdominal wall motion during contractions; some argue this is largely a mechanical artifact rather than true myometrial activity [10]. The fast wave (FW) corresponds to myometrial action potentials and is further divided into a fast-wave-low component (FW_L; ~0.1–0.3 Hz, up to 0.5 Hz), consistently present during contractions, and a fast-wave-high component (FW_H; ~0.5–1.0 Hz, sometimes up to 2–3 Hz), emerging during strong, active labor. Spectral peak frequency typically increases as labor approaches, shifting from ~0.2–0.3 Hz in late pregnancy to ~0.6–0.8 Hz during active labor [15]. This reflects more rapid, synchronized firing of uterine myocytes.

Temporal patterns: In the time domain, EHG during rest shows baseline noise only. Contractions are marked by a slow upward drift (SW) with bursts of higher-frequency oscillations lasting 30–90 s, mirroring contraction duration. Bursts increase in intensity to a peak mid-contraction before declining. In true labor, these events occur at regular intervals, aligning closely with mechanical tocodynamometry traces [15]. Subtle frequency and propagation changes can precede overt labor, suggesting predictive potential.

Propagation and multichannel features: Multichannel studies show that activity often originates near the fundus and spreads downward. Cross-correlation of electrode signals allows estimation of conduction velocity and propagation direction. Reported velocities are on the order of a few centimeters per second, and several studies report an apparent increase as labor approaches [16]. Physiologically, this faster, more organized propagation is attributed less to a change in the intrinsic conduction speed of a single fiber than to increased intercellular coupling: the rise in gap-junction (connexin-43) density and myometrial excitability toward term lets depolarization spread more synchronously across a larger volume [11,12]. Changes in tissue properties such as myometrial thickness, water content, and structural organization may also alter the apparent surface velocity, so estimated values should be read as effective, tissue- and method-dependent quantities rather than a fixed conduction constant. Active labor is associated with highly synchronized, near-global activation across the uterus.

In summary, a typical EHG contraction trace features a low-frequency baseline rise with an embedded burst of ~0.3–1 Hz electrical activity. The frequency content shifts higher, and the burst intensity increases as contractions become stronger and more coordinated (for instance, moving from irregular Braxton Hicks contractions to regular active-labor contractions). These measurable changes in the EHG signal form the basis for various predictive and monitoring applications.

### 2.5. Signal Processing and Feature Extraction

A variety of signal-processing techniques have been applied to EHG signals to extract meaningful features for clinical or research purposes.

Frequency analysis: Spectral analysis (e.g., FFT, power spectral density) provides metrics such as peak frequency, median frequency, and band power. Ratios of high-to-low frequency band energy are commonly used to track labor progression [15].

Amplitude/envelope analysis: Techniques such as RMS amplitude, peak-to-peak voltage, and integrated EMG (IEMG) quantify contraction intensity. While useful, amplitude is sensitive to electrode conditions and is thus rarely used in isolation.

Temporal pattern analysis: Burst detection and inter-contraction interval analysis replicate mechanical contraction patterns. Automated algorithms increasingly combine time and frequency features to identify contractions [17].

Nonlinear and complexity features: Uterine EMG signals are generated by complex, nonlinear physiological processes and often exhibit nonstationary behavior. Hence, nonlinear features have been explored to improve discrimination between pregnancy and labor, or between term and preterm labor. Fractal and entropy measures, including approximate entropy, sample entropy, and correlation dimension, attempt to quantify the regularity or complexity of the EHG signal. As labor approaches, uterine electrical activity tends to become more organized (less random), which may be reflected in decreasing entropy measures. Some studies have found that nonlinear metrics outperform simple linear features in distinguishing laboring from non-laboring signals [17]. Wavelet transforms allow time-frequency analysis of how signal energy in various frequency bands evolves over time within a contraction, capturing the transient nature of contraction signals; wavelet-based features have been used to classify EHG recordings (e.g., distinguishing true vs. false labor) [18].

Propagation and synchronization indices: Cross-correlation and phase synchronization quantify electrical coupling across sites, with increases linked to effective labor contractions [16].

Machine-learning classification: Recent work has applied classifiers (e.g., support vector machines, neural networks) to combined feature sets. Ren et al. (2015) reported an AUC of approximately 0.98 for preterm-birth prediction using entropy features and empirical mode decomposition [17]; however, such figures derive from retrospective analysis of a single public database and require prospective, external validation before clinical use. Integration of large datasets and public EHG repositories is accelerating algorithm development. A recent systematic review has catalogued automatic methods for detecting uterine contractions from EHG before and during labor [19], and machine learning has been used to derive EHG-based digital biomarkers such as cervical-dilation classification [20]. Other recent studies have applied empirical mode decomposition and signal-synchronization measures to discriminate imminent or preterm delivery [21,22]. Deep-learning architectures (including attention-based and hypergraph neural networks), resampling and cross-validation strategies, and multifactorial models combining bioelectrical with clinical parameters have further advanced preterm-birth prediction [23,24,25,26,27,28].

In summary, advances in electrode technology, amplification, filtering, and processing have made EHG acquisition more robust and accessible. Characteristic changes in amplitude, frequency, temporal patterns, and propagation provide valuable biomarkers of uterine activity. Coupled with machine learning, these features hold promise for clinical applications in labor monitoring and preterm-birth prediction.

### 2.6. Clinical Applications of EHG

Because EHG measures the electrical activity that drives contraction, it supports several clinical uses. We order them by nearness to translation and confirmed utility from promising but as-yet-unvalidated applications; the evidence tier of each is made explicit in Section 7 (Table 3).

Intrapartum contraction monitoring (confirmed utility): EHG detects contraction timing and frequency at least as reliably as the tocodynamometer and approaches the intrauterine pressure catheter, with a near one-to-one correspondence between electrical bursts and pressure rises [15]. It outperforms tocodynamometry where the latter is weakest, notably with maternal obesity and movement, showing markedly higher sensitivity and fewer signal-loss events [1,2]. This is the application for which the evidence is strongest.

Preterm-birth prediction and true-versus-false-labor discrimination (promising use): Rising spectral peak frequency and propagation velocity track the approach of true labor and help distinguish it from threatened but non-progressing labor [18]. Recorded weeks in advance, peak frequency, and nonlinear features differ between women who deliver preterm and at term, and a combination of propagation velocity and peak frequency predicted delivery within seven days with an area under the curve of about 0.96, exceeding cervical examination and fetal fibronectin [3]. These results are encouraging but derive mainly from retrospective, single-center cohorts and require prospective validation [29].

Labor dysfunction and tocolytic monitoring (speculative): Abnormally high peak frequencies during early labor have been reported in women who later required cesarean delivery for arrest, possibly reflecting poorly coordinated, ineffective contractions; sustained or high-frequency activity has also been interpreted as a signature of uterine muscle fatigue [30]. Likewise, a fall in EHG activity after tocolysis could in principle gauge treatment response. These uses remain physiologically plausible but clinically unvalidated. More recently, EHG has been used to quantify the effect of labor epidural analgesia on uterine activity [31]. A related pilot study documented changes in uterine contraction frequency after epidural initiation [32].

Telemonitoring (emerging): The noninvasive, surface-based nature of EHG suits outpatient and home monitoring; wearable patches and belts transmit contraction data for remote review and their feasibility for detecting preterm labor signatures earlier than symptoms has been reported [29] (Section 6). Home monitoring uses surface EHG, never invasive electrodes.

### 2.7. Limitations and Challenges of EHG

EHG faces technical and practical barriers. The abdominal signal is small and easily buried in noise from maternal and fetal ECG, respiration, skeletal-muscle EMG, and motion; good recordings require careful skin preparation, stable electrodes, and adaptive filtering, and poor signal-to-noise ratio can cause missed or false contraction detections, especially early in pregnancy [10]. Signals also vary widely between and within women with adipose thickness, electrode placement, parity, and gestational age, so absolute thresholds are hard to define and most work relies on within-subject normalization; body mass index, for example, has been shown to modify maternal cardiac–uterine coupling during labor [33], and uterine electrical activity differs between singleton and multiple gestations [34]. The field further lacks standardized acquisition and analysis, which impedes comparison across studies and is the central theme of Section 7. Motion, coughing, and, in the second stage, maternal pushing produce artifacts in the same low-frequency band as contractions; auxiliary motion sensing helps, but artifact-free ambulatory recording remains difficult. Crucially, EHG reflects excitability and timing but does not directly measure force, so it cannot yet replace Montevideo units for quantifying contraction strength, and translation of electrical features into pressure-equivalent metrics is imprecise. Finally, clinicians are unfamiliar with EHG tracings, integration into monitors and records is incomplete, and, as with any diagnostic test, false positives or negatives carry clinical and ethical costs; large-scale validation and training are prerequisites for routine use.

## 3. Electromyography (EMG) for Uterine Contractility

EMG, in the context of uterine contractility, encompasses techniques that measure the electrical activity generated by uterine smooth muscle. While EHG represents the noninvasive, surface-based form of uterine EMG, a broader view includes invasive recordings, biophysical mechanisms, and comparative electromyographic principles drawn from classical muscle physiology. This section outlines the historical development of uterine EMG, the physiological distinctions between uterine and skeletal muscle activity, and the technical methods used to acquire and interpret uterine EMG signals. In current clinical practice, “uterine EMG” is effectively synonymous with EHG; the invasive and multiscale material below is included to ground interpretation of the surface signal, not as a separate clinical modality.

### 3.1. Historical Development and Context

The study of uterine EMG has evolved alongside the development of EHG. Early investigations in the period of 1920–1940 employed intracellular electrodes or organ-bath setups in animal tissue to record spontaneous uterine electrical oscillations. These foundational experiments established that the uterus generates its own rhythmic depolarizations, laying the groundwork for understanding uterine excitability.

By the 1960s, physiologists such as Arpad Csapo advanced the field through in vivo experiments, placing electrodes on or near the uterus in animal models to correlate electrical activity with contraction force. These studies revealed key hormonal influences, notably that progesterone suppresses uterine electrical excitability [35].

Invasive uterine EMG recordings were later performed in humans during surgical procedures such as cesarean deliveries, offering rare but direct insights into uterine electrophysiology. However, these methods were invasive and carried risks of infection, and so were not clinically practical.

During the 1970s and 1980s, interest shifted toward noninvasive surface recordings such as EHG, while animal studies continued to refine high-fidelity internal EMG recordings using implantable electrodes. Garfield and colleagues, for example, used abdominal surface recordings in pregnant rats to track myometrial EMG activity throughout gestation, establishing a direct link between electrical bursts and contractile activity [36]. Such studies confirmed that internal electromyograms coincide with external EHG signals and intrauterine pressure changes, validating the surface approach [10].

In modern practice, “uterine EMG” generally refers to transabdominal EMG, synonymous with EHG. Nevertheless, understanding the historical and biophysical foundation of invasive EMG is crucial for interpreting surface recordings and for developing models of uterine excitation and propagation.

### 3.2. Physiological Basis of Uterine EMG vs. Skeletal Muscle EMG

Electromyography in the classical sense often refers to recording electrical activity from skeletal muscle. Uterine muscle, being smooth muscle, differs significantly in its electrophysiological behavior. These distinctions shape both the waveform characteristics and the interpretation of uterine EMG.

Excitation mechanism: Skeletal muscle contractions result from somatic motor-neuron stimulation, leading to rapid, all-or-none action potentials. In contrast, uterine smooth muscle is self-excitable, exhibiting intrinsic pacemaker activity modulated by hormonal and paracrine influences. Uterine action potentials are calcium-based plateau spikes with long durations, supporting sustained contractions. Consequently, uterine EMG signals are low-frequency, long-duration bursts rather than the brief spikes typical of skeletal-muscle EMG.

Frequency content: Typical skeletal-muscle EMG (from a biceps or leg muscle, for example) has significant power in the 50–150 Hz range due to short-duration action potentials (2–5 ms) and fast conduction velocities. Uterine muscle action potentials last much longer (50–1000 ms, including plateaus) and propagate slowly, yielding frequency content mainly under 5 Hz. This low-frequency nature demands specialized amplifiers and filters, as conventional EMG systems might otherwise suppress these frequencies as “noise” [10].

Propagation and recruitment: Whereas skeletal muscles consist of independently firing motor units, the uterus behaves as a functional syncytium. Gap junctions between myocytes enable the wave-like propagation of depolarization across large regions during contractions. As a result, uterine EMG waveforms appear as coherent bursts or traveling waves rather than the irregular interference patterns seen in skeletal EMG. During active labor, this synchrony increases markedly, producing stronger, more coordinated electrical bursts [12].

Muscle fiber types: Uterine myocytes are smooth muscle cells with a different ion-channel composition (prominent L-type calcium channels, fewer sodium channels) and no organized sarcomere structure like skeletal muscle fibers. Their slower action potentials and plateau phases yield smoother EMG waveforms. Additionally, subthreshold slow waves can contribute to baseline activity, reflecting the uterus’s readiness for coordinated contraction.

Surface vs. intracellular signals: Intracellular uterine action potentials range from 40 to 80 mV, but surface or extracellular electrodes detect only microvolt-level signals after attenuation through tissue layers. Direct myometrial electrodes record signals of tens of millivolts, comparable to skeletal-muscle needle EMG. Thus, surface EHG captures a far-reduced, filtered representation of the uterine depolarization field. Understanding this distinction is key to interpreting the relatively low amplitudes observed in clinical EHG.

**Figure 2:**
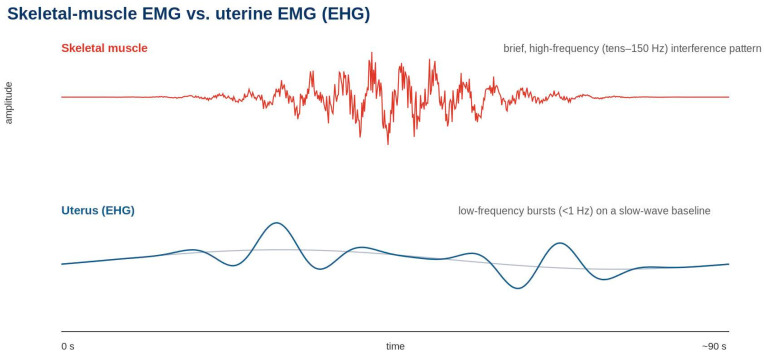
Schematic contrast between skeletal-muscle EMG and uterine EMG (EHG). Skeletal-muscle EMG (top) consists of brief, high-frequency interference-pattern activity (tens to ~150 Hz) generated by asynchronously firing motor units. Uterine EMG (bottom) consists of low-frequency bursts (dominant energy below ~1 Hz) of long-duration, calcium-mediated action potentials that summate as the uterus behaves as an electrical syncytium, superimposed on a slow-wave baseline. The two differ by roughly two orders of magnitude in dominant frequency and time scale.

In summary, the physiological basis of uterine EMG is rooted in smooth-muscle electrophysiology: slower events, synchronous recruitment over large areas, heavy influence of cellular connectivity, and modulation by hormones. These features produce an EMG signal that is qualitatively different from the rapid, segmental EMG of skeletal muscle. Nonetheless, both are manifestations of muscle-membrane depolarization, so the general EMG principles, the need for differential recording, elimination of noise, and interpretation of frequency content, apply, but with modifications appropriate to the uterine context (Figure 2).

### 3.3. EMG Signal Acquisition Techniques (Beyond Surface EHG)

**Figure 3:**
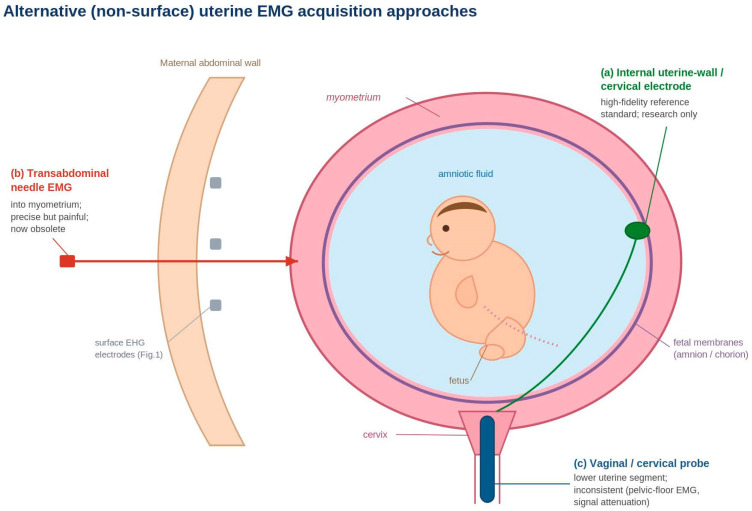
Alternative (non-surface) approaches for acquiring uterine EMG. Sagittal schematic showing the maternal abdominal wall, myometrium, fetal membranes (amnion/chorion), and amniotic fluid. (**a**) Internal electrodes placed on the uterine wall or cervix give high-fidelity, low-noise recordings and serve as the reference standard, but are restricted to research because of infection risk and invasiveness. (**b**) Transabdominal needle electrodes inserted into the myometrium yield precise local signals but are painful and hazardous (infection, uterine irritability, fetal injury) and are now obsolete. (**c**) Vaginal or cervical probes record from the lower uterine segment but give inconsistent results owing to pelvic-floor muscle EMG interference and signal attenuation. Surface EHG electrodes on the abdominal wall (Figure 1) are shown for reference.

Although surface EHG is the primary clinical and research method for recording uterine electrical activity, several other acquisition approaches have been explored, each balancing signal fidelity and invasiveness (Figure 3).

Internal uterine electrodes: In experimental or surgical contexts, electrodes can be placed directly on the uterine wall or cervix, yielding high-fidelity, low-noise recordings. These internal EMGs correlate closely with contraction pressure and are considered the reference standard for validating non-invasive methods. However, in human clinical care, their use is restricted to research due to infection risk and invasiveness.

Needle EMG: Historically, fine-needle electrodes were inserted transabdominally into the myometrium to record local activity. Despite producing strong, precise signals, this approach proved painful and hazardous due to the risk of infection, uterine irritability, and fetal injury. It is now obsolete outside of limited physiological research.

Vaginal/cervical probes: Electrodes placed around the cervix or within the vagina can record activity from the lower uterine segment. These devices have been evaluated for preterm-labor detection, but results are inconsistent due to interference from pelvic-floor-muscle EMG and signal attenuation. Comfort and reproducibility issues further limit clinical feasibility.

Multi-electrode mapping systems: Advanced systems employing dense electrode arrays across the maternal abdomen, such as electromyometrial imaging (EMMI), can reconstruct three-dimensional uterine activation patterns using inverse-modeling techniques [37]. This enables visualization of propagation pathways and synchronization across the uterus. While promising for research, such high-density mapping remains clinically impractical due to its complexity and computational demands.

Instrumentation and filtering differences: Internal electrodes capture broader frequency components (up to 20–30 Hz) than surface EHG because tissue layers act as a natural low-pass filter. Consequently, filtering parameters, amplification needs, and even artifact susceptibility differ between acquisition types. Optimal instrumentation aims to maximize uterine signal sensitivity and minimize contamination from other physiological or environmental sources.

Integration with other sensors: Combining EMG with pressure transducers or strain sensors enables cross-validation of electrical and mechanical activity. Such multimodal systems could eventually provide a comprehensive assessment of uterine function, using EMG to detect excitability and timing while pressure sensors quantify contraction strength.

Uterine EMG represents a powerful window into the bioelectrical mechanism driving labor. From early invasive studies to modern noninvasive EHG, it has illuminated the electrophysiology of uterine contractions and provided a foundation for clinical innovation. Distinct from skeletal-muscle EMG, uterine EMG is characterized by slow, synchronous, calcium-mediated depolarizations that reflect the coordinated excitability of smooth muscle tissue. Continued refinement of acquisition technologies and analytical models promises to enhance our ability to monitor, predict, and manage labor through the lens of uterine electrophysiology.

### 3.4. Characteristics of Uterine EMG Signals (Cellular, Myometrial, and Abdominal Levels)

Uterine EMG manifests differently across biological scales. At the cellular level, single myocytes show rhythmic slow depolarizations with superimposed action-potential spikes recordable by microelectrodes; near term, cells fire regular bursts of long-duration (hundreds of milliseconds), calcium-driven spikes, an intrinsic pacemaker-like rhythmicity not localized to any one cell. At the myometrial-tissue level, contact electrodes record the summation of many myocytes as compound bursts that occur in phase with intrauterine pressure, confirming the electrical activity as the direct precursor of contraction [10]; internal recordings reach the millivolt range with sharp spike morphology and dominant frequencies below ~5 Hz. At the abdominal surface, EHG provides a filtered, lower-amplitude composite (tens to a few hundred microvolts) dominated by 0.3–1 Hz fast-wave energy, with the sharpest high-frequency features attenuated by intervening tissue; nonetheless, internal and surface recordings share timing and spectral profile, validating surface EHG as a reliable proxy [10]. This roughly tenfold amplitude reduction motivates the high-gain, low-noise front ends and robust filtering of surface systems.

Beyond the fast wave, the uterine signal also contains a very-low-frequency slow-wave component (roughly 0.01–0.03 Hz). Its origin is debated: part is generally attributed to mechanical artifact and baseline shifts accompanying contraction, but preclinical and clinical recordings suggest a genuine slow-wave substrate reflecting myometrial tone and excitability, and it has received far less analytical attention than the fast-wave bursts [10]. Characterizing the slow wave, and separating its physiological and mechanical contributions, is an open problem that may add information beyond burst analysis; recent signal-processing work has begun to exploit the relationship between slow- and fast-wave components to improve contraction detection [38].

Spatially, activity typically originates near the fundus and propagates toward the cervix; upper-abdominal leads detect bursts slightly before lower leads, and disorganized or double-peaked propagation may mark labor dysfunction.

### 3.5. Signal Processing and Feature Extraction for Uterine EMG

The analytical pipeline for uterine EMG largely mirrors that described for EHG in Section 2.5: spectral parameters (peak and median frequency, band-power ratios), amplitude and burst metrics, propagation and coherence measures, nonlinear and entropy features, and, increasingly, machine-learning and deep-learning classifiers [17]. Two considerations are specific to the broader EMG setting. First, recording scale dictates the achievable features: internal or animal recordings resolve individual action potentials (spike amplitude, duration, firing rate), whereas surface EHG yields only aggregate envelope and spectral features. Second, because clinical classifiers must ultimately be trusted by clinicians, there is growing emphasis on models whose features are biologically interpretable rather than opaque; nonlinear parameterizations that map onto myometrial excitability and coupling have been proposed to this end [30], and systematic evaluations of artificial intelligence methods for preterm-birth prediction underscore both their promise and the need for explainability and external validation [39]. This solution was presented by Radomski et al., who proposed a classifier based on a clinically interpretable parameter called the uterine activity integral parameter [40]. Public repositories such as the PhysioNet Term-Preterm EHG database continue to enable reproducible modeling [17].

### 3.6. Clinical and Research Applications of Uterine EMG

Most clinical applications of uterine EMG are those already described for EHG in Section 2.6; here we note the perspectives that a broader, including invasive and preclinical, view adds. As a research reference standard, internal uterine EMG has been used to validate external monitors, as electrical activation provides direct evidence that a contraction has occurred. In pharmacological studies, EMG quantifies drug effects objectively: tocolytics (beta-agonists, magnesium sulfate, nifedipine) reduce burst frequency and amplitude, whereas uterotonics increase activity. EMG also offers a window on labor disorders, distinguishing electrically overactive but ineffective (dyscoordinated) contractions from mechanical obstruction, and has been proposed as a means of detecting uterine muscle fatigue [30]. Postpartum, absent or insufficient electrical activity could, in principle, flag uterine atony before hemorrhage; recent work has begun to characterize uterine activity in the first half-hour after childbirth using EHG [41]; postpartum EHG parameters have been positively associated with blood loss after vaginal delivery [42]; and the topic has been surveyed in a recent scoping review [43]. In basic science, implanted electrodes in animal models link ion-channel and hormonal manipulations to contractile output. Of these, only intrapartum contraction detection is established clinically; the pharmacological, fatigue, dystocia, and postpartum uses remain promising but unvalidated (Section 7, Table 3). Home and outpatient monitoring, it bears repeating, is performed with noninvasive surface EHG, not invasive electrodes.

### 3.7. Limitations and Challenges of Uterine EMG

The limitations of uterine EMG overlap substantially with those of EHG (Section 2.7); a few are specific to the broader modality. The defining trade-off is between invasiveness and signal quality: internal electrodes yield clean, high-amplitude recordings but carry infection and injury risk and are confined to research, whereas noninvasive surface recording is safe but attenuated and noise-prone, placing the burden on signal processing. Contamination is a particular challenge because the abdominal surface simultaneously carries maternal and fetal ECG, respiration, skeletal-muscle EMG (notably during maternal pushing, whose energy lies above ~10 Hz), and motion artifacts; adaptive filtering and reference subtraction are needed to recover the low-frequency uterine signal. A less widely appreciated source of overlap is other abdominal smooth muscle: gastrointestinal myoelectrical activity occupies a similar low-frequency range and is recordable from the same abdominal sites, so recent work on separating uterine from gastrointestinal smooth-muscle EMG is directly relevant to obtaining clean uterine recordings [44]. Finally, unfamiliarity, cost, and the still-limited evidence linking EMG-guided management to improved outcomes continue to slow adoption [18].

## 4. Cardiotocography (CTG)

Cardiotocography is the most widely used method in current obstetric practice for assessing fetal well-being and uterine activity. CTG technology comprises electronic devices designed to record, analyze, and display the fetal heart rate (FHR) alongside uterine contraction (UC) activity. CTG is used extensively to assess fetal condition, particularly during the antepartum and intrapartum periods.

### 4.1. Historical Development and Principles

**Figure 4:**
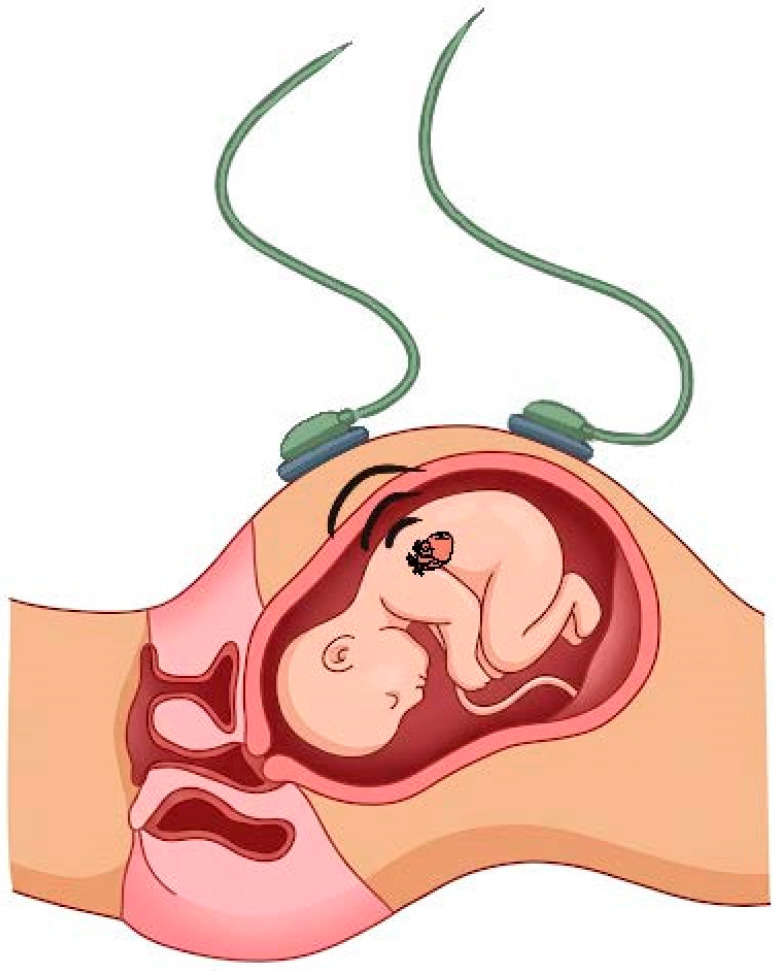
External cardiotocography (CTG). Two transducers are secured to the maternal abdomen with elastic belts: a Doppler ultrasound transducer positioned over the fetal heart records the fetal heart rate, while a tocodynamometer placed near the uterine fundus registers uterine contractions as changes in abdominal-wall tension. The tocodynamometer therefore reflects the mechanical consequence of contraction rather than myometrial electrical activity, and its performance degrades with high maternal body-mass index and with maternal movement.

The development of CTG began in the late 1950s and early 1960s, when several researchers independently contributed to its foundation. Hon in the United States, Caldeyro-Barcia in Uruguay, and Hammacher in Germany each introduced key concepts for the continuous monitoring of fetal heart rate and uterine activity [45]. Their combined work established cardiotocography as a clinical method. CTG was originally developed to help obstetricians and midwives identify changes in fetal heart rate during labor and intervene promptly to prevent intrapartum hypoxic-ischemic injury. Although it is widely used as a screening tool for fetal hypoxia, its positive predictive value is relatively low, approximately 30%. We include CTG because it is the current clinical standard and the comparator against which EHG must be judged; the fetal-heart-rate detail below is limited to what is needed to contrast its uterine-activity channel with electrical methods (Figure 4).

### 4.2. Signal Acquisition

For external monitoring of FHR, an ultrasound transducer is used. This device contains piezoelectric crystals that convert electrical oscillations into ultrasound waves via electroacoustic coupling and then receive the reflected signals. It operates on the Doppler principle: when ultrasound waves encounter moving structures of spatially varying acoustic impedance, such as components of the fetal heart, they are reflected with a corresponding change in frequency. This frequency shift is detected and processed by the piezotransducer. The resulting signal originates from the movement of intracardiac structures, including the cardiac valves and the interventricular septum; both continuous and pulsed Doppler techniques may be applied [45]. The transducer is placed on the maternal abdomen and secured with an elastic belt, while conductive gel ensures efficient transmission of ultrasound waves through proper acoustic-impedance matching.

From approximately 26–28 weeks of gestation onward, it becomes possible to obtain more consistent and reliable signals, a characteristic which has contributed to the rapid adoption of CTG in clinical practice. However, the technique has several noteworthy limitations. The recorded signal may be affected by movements of other non-relevant intra-abdominal structures, such as maternal blood vessels or fetal limbs, leading to artifacts. Early attempts to reduce these artifacts by filtering signals beyond a certain ultrasound-beam depth have not been sufficiently effective [45]. In addition, the signal does not always present an unambiguous peak in reflection amplitude, which complicates accurate measurement of interbeat intervals.

### 4.3. Signal Processing and Generations of Monitors

Early cardiotocographs used intensity-based peak detection and were prone to noise and artifact. Second-generation monitors added wider ultrasound beams and digital processing, chiefly autocorrelation, which compares the incoming Doppler signal with a stored segment of several cardiac cycles to estimate the beat interval [46]. This reduced signal loss and produced tracings closer to those from a fetal scalp electrode [47,48], but autocorrelation yields only an approximate interbeat interval and remains vulnerable to signal loss and to inadvertently recording the maternal rather than the fetal heart rate [45]. An alternative that avoids these Doppler limitations is extraction of the fetal ECG from maternal abdominal electrodes, the same electrodes that record the EHG, allowing fetal heart rate and uterine activity to be obtained together [14]. Uterine contractions themselves are registered by the tocodynamometer, which senses abdominal wall tension rather than intrauterine pressure and is correspondingly sensitive to body habitus and maternal position.

## 5. Comparative Analysis: EHG, EMG, and CTG for Uterine Contractility

Electrohysterography, invasive/internal uterine EMG, and cardiotocography record different quantities and serve different purposes; Table 1 summarizes the comparison. EHG and internal uterine EMG measure the same physiological event, myometrial electrical activation, but differ in access and fidelity. Surface EHG is entirely noninvasive and is the form used in all routine and home settings; internal (intrauterine or intramyometrial) EMG is of a higher fidelity but invasive and restricted to research or intraoperative use. To avoid ambiguity, throughout this review “EHG” denotes noninvasive surface uterine EMG and “EMG” denotes invasive uterine internal recording; there is no separate “noninvasive EMG” distinct from EHG. Despite attenuation, surface EHG correlates closely with internal EMG and intrauterine pressure in timing and spectrum [10], and detects contractions far more sensitively than the tocodynamometer: in laboring women, EHG identified more than 90% of intrauterine-pressure-catheter contractions versus about 54% for tocodynamometry [1], the advantage being most marked at a high body mass index [2].

Cardiotocography addresses a partially different question. Its tocodynamometric channel senses only abdominal wall tension, so it is the least accurate of the three for contraction detection and degrades with obesity and movement; however, CTG uniquely provides fetal surveillance through the fetal heart rate, which the electrical methods do not. Importantly, the same abdominal electrodes used for EHG can also recover the fetal ECG, so a single noninvasive electrode set can, in principle, yield both uterine activity and fetal heart rate [14]; this convergence, rather than direct competition, is the likely role of EHG alongside fetal monitoring. In the frequency domain, internal EMG can contain components to 20–30 Hz, surface EHG is confined below ~5 Hz by tissue filtering, and the toco is a slow mechanical signal, so analysis settings must match the acquisition context.

In short, EHG offers the best balance of safety, accuracy, and versatility for uterine-activity monitoring, approaching invasive fidelity without its risks and outperforming tocodynamometry, while CTG remains necessary for the fetal component it alone provides.

## 6. Emerging Wearable and Portable Technologies for Uterine Activity Monitoring

Because EHG is acquired from surface electrodes, it is well suited to wearable, wireless implementation, extending uterine monitoring from the labor ward to the home. Adhesive patch sensors integrate electrodes, amplifier, and a wireless link into thin, conformable devices for daily or overnight wear, and flexible electrode designs improve comfort and signal pickup for long-term recording [49]. Smart belt systems embed EHG electrodes together with accelerometers and ECG channels; the FDA-cleared INVU (Nuvo) belt, for example, records fetal and maternal heart rate and uterine activity and streams data for remote review, with abdominal electrodes recovering the fetal ECG alongside EHG [14]. A recent prospective cohort reported good patient acceptability of intrapartum monitoring based on wireless transabdominal fetal ECG and EHG [50]. Pilot telemonitoring programs give high-risk patients such devices to wear during the third trimester, with automated analysis alerting clinicians to changes suggestive of early labor, and feasibility for detecting preterm-labor signatures before symptom onset has been reported [29].

These systems increasingly pair continuous acquisition with cloud-based machine learning that flags trends (rising contraction frequency or peak frequency) rather than raw data, functioning as early-warning systems, and with app interfaces that support patient engagement while avoiding undue anxiety. Practical priorities are energy efficiency (multi-day battery life, low-power or disposable patches), skin-safe long-wear adhesives, secure encrypted data handling, and integration with electronic health records. Realized at scale, wearable EHG could shift preterm-labor management from reactive to proactive; its ultimate value, like that of the modality generally, will depend on the standardization and validation discussed next.

## 7. Toward Standardization: A Proposed Minimum Reporting Framework

A recurring theme across this review, and arguably the single greatest obstacle to clinical translation, is the absence of standardized acquisition and reporting in EHG research. Earlier authors have likewise called for greater standardization and have reviewed acquisition choices [29]; our aim is to consolidate these into a single, checkable minimum reporting set and to extend it explicitly to analysis and clinical validation. Studies differ in electrode material, number, configuration, and inter-electrode distance; in analogue and digital filter settings; in the frequency bands and mathematical definitions used for features; and in the gestational ages, populations, and outcome definitions studied. This heterogeneity makes published performance figures difficult to compare and effectively precludes the pooled or meta-analytic synthesis that regulatory qualification ultimately requires. Synthesizing the methodological detail surveyed in the preceding sections, we propose a minimum reporting set for EHG studies (Table 2). The intent is not to mandate a single protocol, as the optimal configuration remains an open research question, but to ensure that every study reports the parameters needed for its results to be interpreted, reproduced, and compared. We group the recommended items into three domains: acquisition, signal processing, and clinical or validation context.

Acquisition. Reports should specify electrode type and material (for example, disposable Ag/AgCl), the number and geometry of electrodes (bipolar pair, grid, or concentric ring), inter-electrode distance, anatomical placement relative to the fundus and midline, reference and ground placement, skin preparation, amplifier gain and input impedance, sampling rate, and analog anti-aliasing filtering.

Signal processing. Reports should state the digital band-pass limits and filter type, powerline and maternal ECG removal methods, artifact-rejection criteria (including any auxiliary motion sensing), the contraction or burst segmentation method, and the precise mathematical definition of every extracted feature together with the frequency band over which it is computed.

Clinical and validation context. Reports should specify gestational age at recording, maternal characteristics (parity, body mass index, singleton or multiple gestation), labor status, the reference standard used (intrauterine pressure catheter, tocodynamometry, or delivery outcome), the outcome definition and time horizon (for example, delivery within seven days), the validation scheme (independent test set versus cross-validation), and performance metrics reported with confidence intervals. Reports should also pre-specify confounders (gestational age, body mass index, parity, electrode placement) and, where machine learning is used, favor explainable, biologically interpretable models rather than opaque classifiers [30,39].

Adoption of even a subset of these items would substantially improve reproducibility, enable the multi-center data pooling on which regulatory qualification depends, and accelerate the maturation of EHG from a promising signal into a validated clinical measurement. Representative clinical and technical studies, summarized in Table 3, illustrate both the consistent promise of the modality and the methodological heterogeneity that a shared reporting standard would address.

**Table 3:** Selected key clinical and technical studies of EHG/uterine EMG.

Study	Population/Design	Modality and Features	Key Reported Performance
Steer 1950 [6]	Human labor	First abdominal EHG recording	Established feasibility (qualitative)
Devedeux 1993 [10]	Review (animal + human)	Internal vs. surface EMG	Internal and surface EMG in phase with IUP; similar spectra
Buhimschi 1997 [51]	40 women, 20–43 wk	Abdominal surface EMG, power spectrum	Activity low through pregnancy, rises sharply at labor
Lucovnik 2011 [3]	116 women	Propagation velocity + PS peak frequency	Delivery <7 d predicted, AUC 0.96 (vs. Bishop 0.72)
Lucovnik 2011 [18]	Term/preterm cohorts	EMG labor diagnosis	Distinguished labor from non-labor
Ren 2015 [17]	TPEHG public database	EMD + sample entropy, ML	Preterm prediction AUC up to 0.99
Euliano 2013 [2]	Laboring women	EHG vs. TOCO vs. IUPC	EHG sensitivity 86–95%; superior at high BMI
Hadar 2015 [1]	43 women, 385 tracings, term	EUM vs. TOCO vs. IUPC	EUM sensitivity 94% vs. TOCO 54%; EUM approx. IUPC
Cohen 2012 [14]	Multicenter, term labor	Abdominal fetal ECG + uterine (AN24)	Abdominal FHR positive agreement ~82% vs. scalp
Garcia-Casado 2018 [29]	Review	EHG for preterm diagnosis	Synthesizes predictive EHG parameters

### Translational Readiness

It is useful to separate what EHG can do now from what remains aspirational. Current applications fall into three tiers of evidence. Category A, supported by clinical evidence, comprises intrapartum detection of contraction timing and frequency, where it matches the performance of intrauterine pressure catheter and outperforms tocodynamometry, including in patients with obesity (Table 3). Category B, promising but not yet validated for routine care, comprises prediction of spontaneous preterm birth and discrimination of true from false labor using spectral peak frequency, propagation velocity, and nonlinear features, where reported accuracy is high but is derived largely from retrospective, single-center cohorts. Category C, experimental or conceptual, comprises tocolytic-response monitoring, postpartum atony surveillance, three-dimensional electromyometrial imaging, and therapeutic electrical modulation of the uterus.

**Figure 5:**
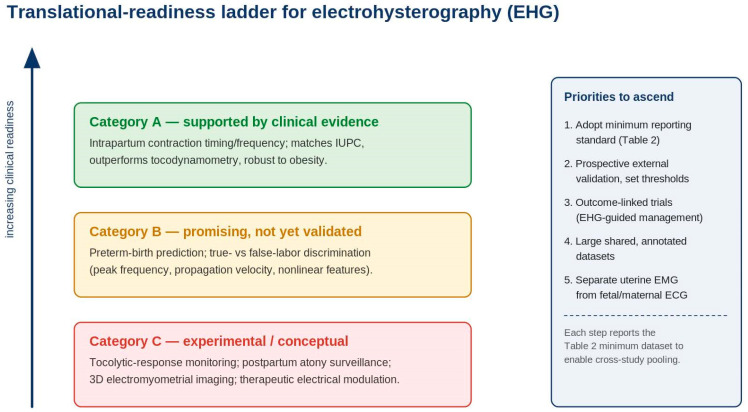
Translational-readiness ladder for electrohysterography. Applications are grouped by evidence tier: Category A, supported by clinical evidence (intrapartum contraction detection); Category B, promising but not yet validated (preterm-birth prediction and true- versus false-labor discrimination); and Category C, experimental or conceptual (tocolytic-response monitoring, postpartum atony surveillance, electromyometrial imaging, and therapeutic electrical modulation). Ascending the ladder depends on five field priorities: adoption of a common minimum reporting standard, prospective externally validated and biologically interpretable prediction models, outcome-linked trials, large shared annotated datasets, and device designs that cleanly separate the uterine EMG channel from fetal and maternal ECG.

Five priorities would move the field from Category B toward Category A: (1) adoption of a common minimum reporting standard (Table 2); (2) prospective, externally validated, and biologically interpretable prediction models with pre-specified decision thresholds and explicit handling of confounders; (3) randomized or pragmatic trials linking EHG-guided management to maternal and neonatal outcomes; (4) large, shared, well-annotated datasets spanning gestational ages and populations; and (5) device designs that cleanly separate the uterine EMG channel from fetal and maternal ECG. These applications and priorities are placed on a research-to-clinic translational ladder (Figure 5).

## 8. Summary and Future Directions

### 8.1. Key Messages

- EHG and invasive uterine EMG measure the electrical driver of contraction directly, unlike the mechanical surrogates used by tocodynamometry and CTG.
- EHG detects contractions far more reliably than tocodynamometry (~94% vs. ~54% against the IUPC) and is robust to maternal obesity.
- Peak frequency and propagation velocity carry preterm-birth predictive information unavailable to mechanical monitors, although current evidence is largely retrospective.
- The chief obstacle to adoption is methodological heterogeneity; a minimum reporting standard (Table 2) is proposed to address this.
- CTG remains essential for fetal surveillance; EHG is positioned to complement or replace its tocodynamometric channel.

### 8.2. Summary

Electrohysterography and uterine electromyography provide powerful windows into the electrical activation of the uterus, the engine behind labor contractions. Historically, researchers have recognized that the key to understanding and predicting labor lies in the uterus’s electrical signals [10]. Over decades, technology advanced from invasive recordings to today’s noninvasive EHG, allowing these signals to be captured safely. We reviewed how EHG/EMG signals originate from smooth-muscle action potentials, propagate via a coupled uterine syncytium, and manifest as low-frequency bursts correlating with contractions. Signal acquisition is feasible with surface electrodes and specialized amplifiers, and the signals typically show a composite of slow baseline shifts and fast-wave components up to ~1 Hz. A multitude of signal-processing techniques, from spectral analysis to machine learning, have been applied to extract features such as peak frequency, which rises as labor nears, or signal entropy, which falls as contractions become more organized. Clinically, uterine EMG has shown value in accurately detecting contractions, distinguishing true from false labor, predicting impending preterm delivery, and characterizing labor efficiency. It holds advantages over traditional tocography, especially in cases of maternal obesity or preterm-labor diagnosis where mechanical methods falter. EHG-based monitors have demonstrated equivalent or superior performance to intrauterine pressure catheters in identifying contraction patterns [1], without the need for uterine invasion. CTG, while limited as a uterine-activity monitor, remains indispensable for its concurrent assessment of fetal well-being. Yet challenges remain, notably, ensuring high signal quality amid noise, standardizing methodologies, and educating clinicians on interpretation. Nevertheless, the momentum is clearly toward incorporating uterine EHG into obstetric practice, as evidenced by emerging FDA-cleared devices and a growing literature supporting its utility.

### 8.3. Future Directions

The near-term agenda follows directly from the priorities set out under Translational Readiness (Section Translational Readiness). Clinically, the field needs practice guidelines and, above all, prospective multi-center and randomized trials that link EHG-guided decisions, for example in preterm-labor triage, to maternal and neonatal outcomes rather than to diagnostic accuracy alone, with one such trial comparing noninvasive electrophysiological monitoring with conventional monitoring during labor is already under way [52]. Such trials should pre-specify thresholds and account for confounders known to affect the signal, including gestational age, body mass index, adipose thickness, parity, and electrode placement. Analytically, larger shared datasets and rigorous external validation are needed, and predictive models should favor biologically interpretable, explainable features over opaque classifiers so that clinicians can understand and trust their output [30,39]. Personalized baselines, in which a woman’s own early-pregnancy EHG calibrates later thresholds, may help manage interpatient variability.

Technically, continued miniaturization, dry electrodes, longer battery life, and multimodal patches that combine uterine EMG with fetal ECG (Section 6) will ease home use. Higher-density and imaging approaches, notably electromyometrial imaging, are moving from animal models to human studies and offer three-dimensional maps of uterine activation and maturation [37,53]. Physiologically, better characterization of pacemaker behavior, of the slow-wave component, and of the separation of uterine from gastrointestinal smooth-muscle activity [44] would deepen interpretation and could eventually support therapeutic modulation of uterine excitability. Because EHG is inexpensive once developed, robust low-power devices could also extend obstetric monitoring to low-resource settings. Across all of these, adoption of a common reporting standard (Section 7) is the enabling step.

## Figures and Tables

**Table 1:** Comparison of uterine monitoring modalities: EHG, EMG, and CTG.

Parameter	EHG	Invasive/Internal Uterine EMG	CTG
Basic principle	Records electrical activity of uterine muscle via surface abdominal electrodes	Direct measurement of myometrial electrical activity using internal electrodes	Measures fetal heart rate (ultrasound) and uterine contractions via external pressure on abdominal wall (toco)
Type of signal	Electrical	Electrical (high fidelity)	Mechanical + Doppler
Invasiveness	Noninvasive	Invasive (intrauterine/intramyometrial)	Noninvasive
Clinical use	Emerging clinical + research	Primarily research	Routine clinical standard care
Accuracy (contractions)	High (>90% vs. IUPC)	Very high (gold standard)	Moderate (~54% vs. IUPC)
Fetal monitoring	No	No	Yes
Physiological insight	Direct uterine electrophysiology	Detailed cellular-level electrical activity	Indirect (mechanical outcome)
Signal quality	Moderate (tissue attenuation)	High	Variable (affected by BMI, motion)
Frequency range	<5 Hz	Up to 20–30 Hz	Low-frequency mechanical
Artifacts	Motion, electrode placement	Less motion artifact but invasive issues	Motion, positioning, obesity
Safety	Very safe	Procedural risk (infection, injury)	Very safe
Ease of use	Moderate	Complex	Easy
Use across pregnancy	Antepartum to intrapartum, outpatient	Limited settings	Mostly intrapartum
Predictive value	Promising for preterm/labor progression	High (research)	Low for hypoxia (~30%)
Cost and availability	Moderate, emerging	High, limited	Widely available, lower cost
Main advantages	Noninvasive, sensitive, physiological insight	Highest fidelity	Widely used, fetal + uterine monitoring
Main disadvantages	Needs standardization	Invasive, not practical	Low specificity, indirect measurement

**Table 2:** Proposed minimum reporting set for electrohysterography (EHG) studies.

Domain	Parameter	Recommended Reporting Detail
Acquisition	Electrode type and material	e.g., disposable Ag/AgCl; gelled vs. dry
Acquisition	Number and configuration	Bipolar pair/grid (e.g., 4 × 4)/concentric ring
Acquisition	Inter-electrode distance	Report exact value (e.g., 2.5–7 cm)
Acquisition	Placement	Relative to fundus, midline, umbilicus
Acquisition	Reference and ground	Anatomical location (e.g., hip)
Acquisition	Skin preparation	Abrasion, gel, impedance check
Acquisition	Amplifier	Gain, input impedance, CMRR
Acquisition	Sampling and anti-alias filter	Sampling rate; analogue low-pass cutoff
Signal processing	Band-pass filter	Cutoffs (e.g., 0.1–4 Hz) and filter type
Signal processing	Interference removal	50/60 Hz notch; maternal-ECG cancellation
Signal processing	Artifact handling	Criteria; auxiliary accelerometer if used
Signal processing	Segmentation	Burst/contraction detection method
Signal processing	Feature definitions	Exact formula + frequency band per feature
Clinical/validation	Gestational age	Weeks at recording
Clinical/validation	Population	Parity, BMI, singleton/multiple
Clinical/validation	Labor status	Term/preterm; labor/non-labor
Clinical/validation	Reference standard	IUPC/tocodynamometry/delivery outcome
Clinical/validation	Outcome and horizon	e.g., delivery <7 days
Clinical/validation	Validation scheme	Independent test set vs. cross-validation
Clinical/validation	Performance metrics	Sensitivity/specificity/AUC + 95% CI

## Data Availability

No new data were created or analyzed in this study. Data sharing is not applicable to this article.
